# Supplementary material for: Genetic variation in the immunoglobulin heavy chain locus shapes the human antibody repertoire
Source: Nat Commun. 2023 Jul 21;14:4419. doi: 10.1038/s41467-023-40070-x (PMC10362067; doi:10.1038/s41467-023-40070-x)
Supplement: Supplementary file 1 — Supplementary Information [file 41467_2023_40070_MOESM1_ESM.pdf]

Supplementary information

Genetic variation in the immunoglobulin heavy chain locus shapes the human antibody repertoire

Oscar L. Rodriguez[1], Yana Safonova[2], Catherine A. Silver[1], Kaitlyn Shields[1], William S. Gibson[1], Justin T. Kos[1], David Tieri[1], Hanzhong Ke[3], Katherine J. L. Jackson[4], Scott D. Boyd[5], Melissa L. Smith[1,*], Wayne A. Marasco[3,*], and Corey T. Watson[1,*]

[1]Department of Biochemistry and Molecular Genetics, University of Louisville School of Medicine, Louisville, KY, USA

[2]Department of Computer Science, Johns Hopkins University, Baltimore, MD, USA

[3]Department of Cancer Immunology and Virology, Dana-Farber Cancer Institute, Harvard Medical School, Boston, MA, USA

[4]The Garvan Institute of Medical Research, Darlinghurst, NSW, Australia

[5]Department of Pathology, Stanford University School of Medicine, Stanford, CA, USA

*To whom correspondence should be addressed

corey.watson@louisville.edu

Wayne_Marasco@dfci.harvard.edu

ml.smith@louisville.edu

Table of contents

Genetic variation in the immunoglobulin heavy chain locus shapes the human antibody repertoire 1

Supplementary Notes 3

1. Detailed descriptions of structural variants 3

2. Biased discovery of novel alleles in self-reported non-White individuals 5

Supplementary Tables 6

Supplementary Table 1. Sequencing statistics across SMRT sequencing systems. 6

Description of Supplementary Figures 7

Supplementary Figure 1. PacBio sequencing and assembly statistics. 8

Supplementary Figure 2. Number and length of merged reads for each AIRR-seq dataset after processing. 10

Supplementary Figure 3. Large SVs with novel SV alleles. 11

Supplementary Figure 4. SNVs genotyped as hemizygous. 12

Supplementary Figure 5. Number of SNVs in different gene components across all IGHV genes. 13

Supplementary Figure 6. Examples of polymorphic indels and small SVs. 14

Supplementary Figure 7. Number of novel alleles per gene. 15

Supplementary Figure 8. Gene usage QTL statistics for the IgG repertoire. 17

Supplementary Figure 9. Comparison of the IgM and IgG gene usage association results. 19

Supplementary Figure 10. Gene usage for genes in the largest deletion identified. 20

Supplementary Figure 11. Gene usage for individuals with variable *IGHV3-23* gene copy number. 21

Supplementary Figure 12. Gene usage for *IGHD3-10* is associated with the IGHD gene region deletion. 22

Supplementary Figure 13. Example of guQTL conditional analysis for the gene *IGHV3-66*. 23

Supplementary Figure 14. Network of all genes connected by shared guQTLs. 25

Supplementary Figure 15. Cliques found in the network. 30

Supplementary References 31

Supplementary Notes

1. Detailed descriptions of structural variants

The two largest SV alleles occurred within a single mSV and were 259 Kbp and 284 Kbp long, resulting in deletions of 14 and 16 IGHV genes, respectively (Fig. 1a; Supplementary Figure 3a). These deletions were observed only in White individuals (n=7). This observation is likely explained by the fact that one of the segmental duplication blocks that mediates these deletions occurs on a complex SV allele with genes *IGHV3-64D* and *IGHV5-10-1*, which is found at higher frequencies in European populations[1]. These large deletions have been partially resolved from AIRR-seq data[2], giving further support to their authenticity.

The region surrounding *IGHV3-30* and *IGHV4-28* and related genes (*IGHV4-30-2, IGHV3-30-3, IGHV4-30-4, IGHV3-30-5, IGHV4-31* and *IGHV3-33*) has been identified previously as a SV hotspot[3]. In earlier studies, 4 SV alleles in this region were fully resolved[3,4]. The longest resolved SV allele spans ~100 Kbp and harbors 4 ~25 Kbp segmental duplications, consisting of repeating IGHV4 and IGHV3 gene cassettes. In this study, we observed 4 of the previously characterized SV alleles, as well as 8 novel SV alleles (Fig. 1a; Supplementary Figure 3b). Relative to the longest SV allele, the other 11 SV alleles contained deletions that varied by position and ranged in size from 23.9 to 74.2 Kbp.

The other SV hotspot identified was an mSV with 4 SV alleles spanning 136 Kbp and included the genes *IGHV4-38-2*, *IGHV3-43D, IGHV3-38-3, IGHV1-38-4, IGHV4-39* and *IGHV3-43* (Fig. 1a; Supplementary Figure 3c). The SV allele harboring these genes is present in our custom reference and was previously resolved[3]. In addition to this haplotype, we identified three deletions (two novel) and one insertion containing two newly discovered paralog genes with 100% sequence identity to *IGHV4-38-2*02* and *IGHV3-43D*03*. Self-alignment of the haplotype

with the insertion to itself further identified that the ~52.2 Kbp insertion is a partial duplication of a previously resolved SV allele[3]. Additionally, we employed adaptive ("read-until") nanopore sequencing in combination with the targeted HiFi long-read sequencing derived assemblies to fully span this event.

In addition to the previously characterized SV allele including *IGHV3-23* and *IGHV3-23D*, we identified a duplication that contained three *IGHV3-23* gene copies (Fig. 1a; Supplementary Figure 3d). Out of the 6 individuals carrying this duplication, 5 were Asian. A higher *IGHV3-23* gene copy number in Asians was reported previously in an early restriction fragment length polymorphism study[5].

While many SVs have been characterized in the IGHV gene region, SVs within the IGHD gene region have usually been predicted using AIRR-seq data, with very limited evaluation by germline locus sequencing[6,7]. Critically, IGHD genes make up a large portion of the complementary determining region 3 (CDR3), the most somatically variable Ab region[8] and a critical determinant of antigen specificity[9]. In our cohort, we characterized a previously inferred deletion spanning 9.6 Kbp, deleting 6 (*IGHD2-8, IGHD1-7, IGHD6-6, IGHD5-5, IGHD4-4, and IGHD3-3*) out of the 26 (23%) IGHD genes (Fig. 1a; Supplementary Figure 3e). Interestingly, this deletion was common (allele frequency = 0.18), present in 23 out of 76 individuals for which genotyping was possible, and homozygous in 5 individuals. One of the homozygotes was also heterozygous for the largest 284 Kbp deletion in the IGHV gene region (Fig. 1d). Just between these two SVs, this individual carried a unique IGH haplotype with 6 deleted IGHD genes and 16 IGHV deleted genes. Taking into account the other SVs concurrently observed, an additional 13 IGHV genes were deleted, totaling 35 deleted IGHV and IGHD genes across both haplotypes in this individual (Fig. 1d).

2. Biased discovery of novel alleles in self-reported non-White individuals

The number of novel alleles identified across the cohort was not equally distributed among individuals. The majority of individuals (n=125; 81%) contained at least one novel allele, with 76 (61%) individuals having 1 to 3 novel alleles. Of the 8 individuals who had 10 or more novel alleles, 5 self-reported as Black or African American. Of the 35 individuals who had 5 or more novel alleles, 14, 7, 4, 2 and 6 were Black or African American, White, South Asian, East Asian and Hispanic or Latino, respectively. This corresponds to 70%, 8%, 20%, 18% and 32% of individuals from each respective subgroup. Furthermore, of the 25 novel alleles found in 5 or more individuals, 3 were found specifically in one subgroup. These novel alleles corresponded to genes *IGHV3-30-3*, *IGHV1-38-4* and *IGHV1-69D*, which are all found within SVs. Additionally, each of these novel alleles appeared at a high frequency, with the novel alleles for *IGHV3-30-3*, *IGHV1-38-4* and *IGHV1-69D* found in 8 Asian, and 7 and 5 Black or African American individuals, respectively.

Supplementary Tables

Supplementary Table 1. Sequencing statistics across SMRT sequencing systems

**System**	**# of individuals**	**Mean polymerase passes (range)**	**Mean expected HiFi read quality (range)**	**Mean IGH HiFi coverage (range)**	**Plex per SMRT cell**
RSII	40	6.0 (3.4 - 12.3)	97.6% (95.9% - 98.7%)	48.7 (6.1 - 90.8)	Single
Sequel	40	19.2 (12.1 - 23.9)	99.9% (98.9% - 99.9%)	40.8 (7.1 - 92.6)	Multi
Sequel IIe	74	21.4 (15.3 - 51.9)	99.9% (99.9% - 99.9%)	109.5 (2.4 - 331.7)	Multi

Description of Supplementary Figures

Supplementary Figure 1. PacBio sequencing and assembly statistics

Supplementary Figure 2. Number and length of merged reads for each AIRR-seq dataset after processing.

Supplementary Figure 3. Large SVs with novel SV alleles.

Supplementary Figure 4. SNVs genotyped as hemizygous.

Supplementary Figure 5. Number of SNVs in different gene components across all IGHV genes.

Supplementary Figure 6. Examples of polymorphic indels and small SVs.

Supplementary Figure 7. Number of novel alleles per gene.

Supplementary Figure 8. Gene usage QTL statistics for the IgG repertoire.

Supplementary Figure 9. Comparison of the IgM and IgG gene usage association results.

Supplementary Figure 10. Gene usage for genes in the largest deletion identified.

SupplementaryFigure 11. Gene usage for individuals with variable *IGHV3-23* gene copy number.

Supplementary Figure 12. Gene usage for *IGHD3-10* is associated with the IGHD gene region deletion.

Supplementary Figure 13. Example of guQTL conditional analysis for the gene *IGHV3-66*.

Supplementary Figure 14. Network of all genes connected by shared guQTLs.

Supplementary Figure 15. Cliques found in the network.

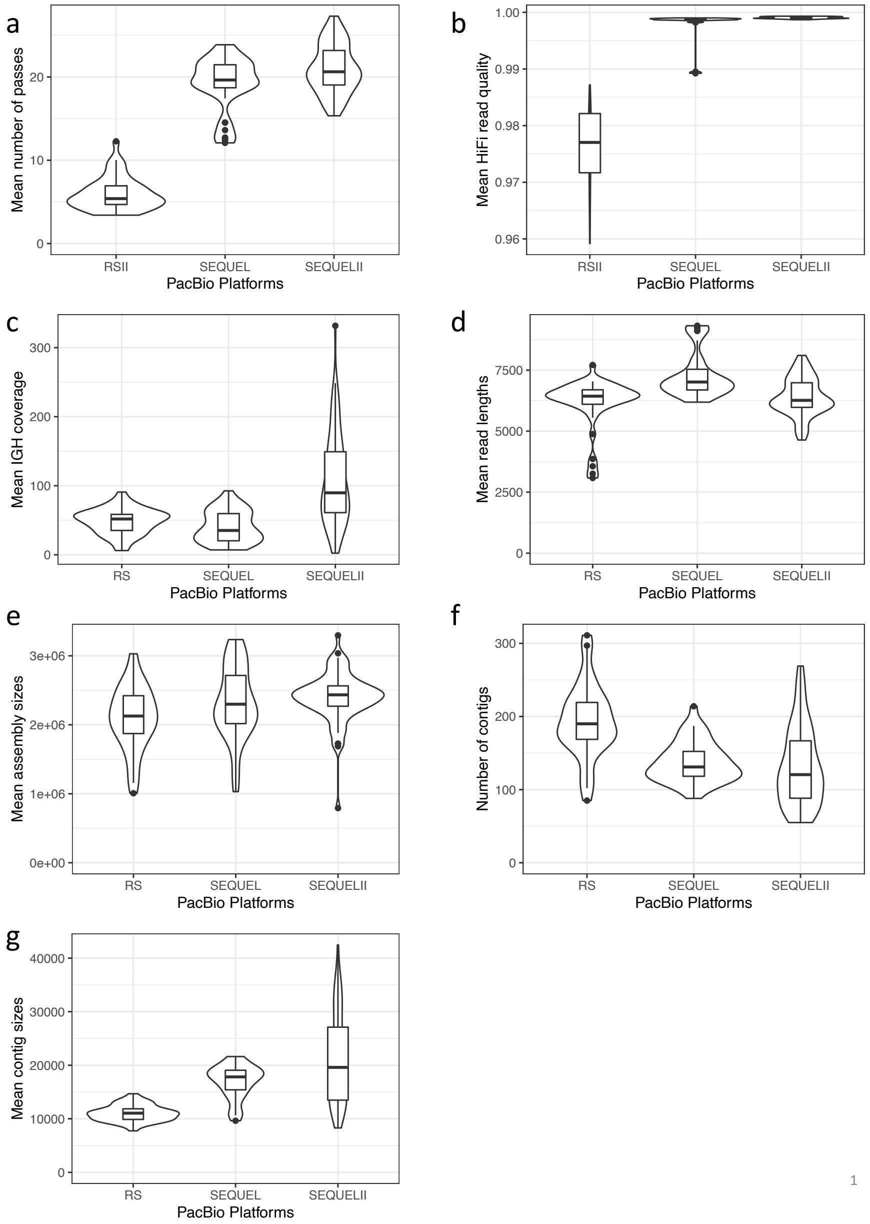

Supplementary Figure 1. PacBio sequencing and assembly statistics.

The statistics described include (a) number of polymerase passes, (b) read quality, (c) IGH locus coverage, (d) read length, (e) assembly sizes, (f) number of contigs and (g) contig sizes. Each statistic is separated by PacBio platforms. The number of sequencing runs on the RS, Sequel, and Sequel II platforms is 40, 40, and 74, respectively. Boxplots display the median,

25th percentile, 75th percentile, and whiskers that extend up to 1.5 times the inter-quartile range (IQR) from the respective percentiles. Any data points outside the whiskers are also plotted.

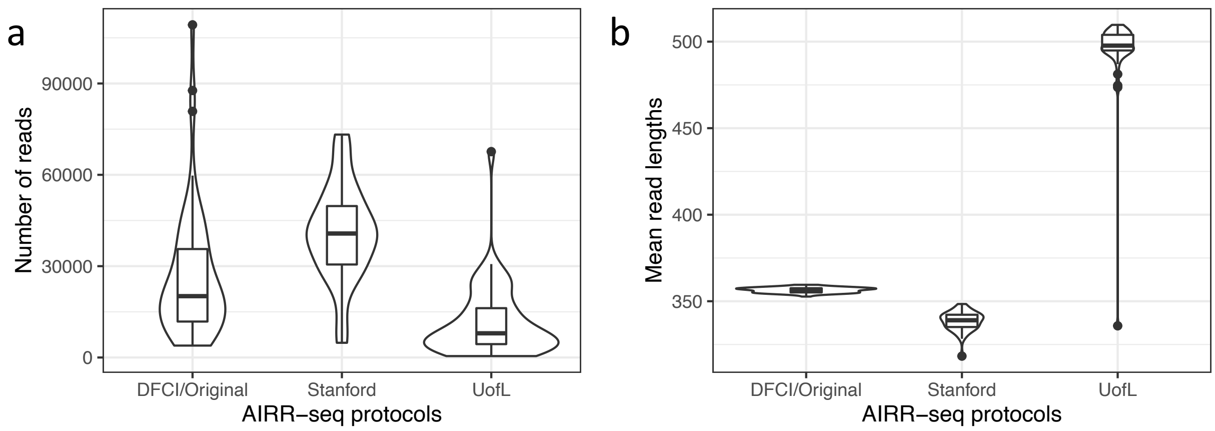

Supplementary Figure 2. Number and length of merged reads for each AIRR-seq dataset after processing.

(a) Number of reads for different AIRR-seq protocols. **(b)** After processing, the AIRR-seq reads are merged. Due to the difference between AIRR-seq protocols, the mean merged read lengths differ between AIRR-seq protocols. The number of sequencing datasets from the DFCI/Original (V-primers), Stanford (V-primers), and UofL (5' RACE) AIRR-seq protocols is 56, 47, and 51, respectively. Boxplots display the median, 25th percentile, 75th percentile, and whiskers that extend up to 1.5 times the inter-quartile range (IQR) from the respective percentiles. Any data points outside the whiskers are also plotted.

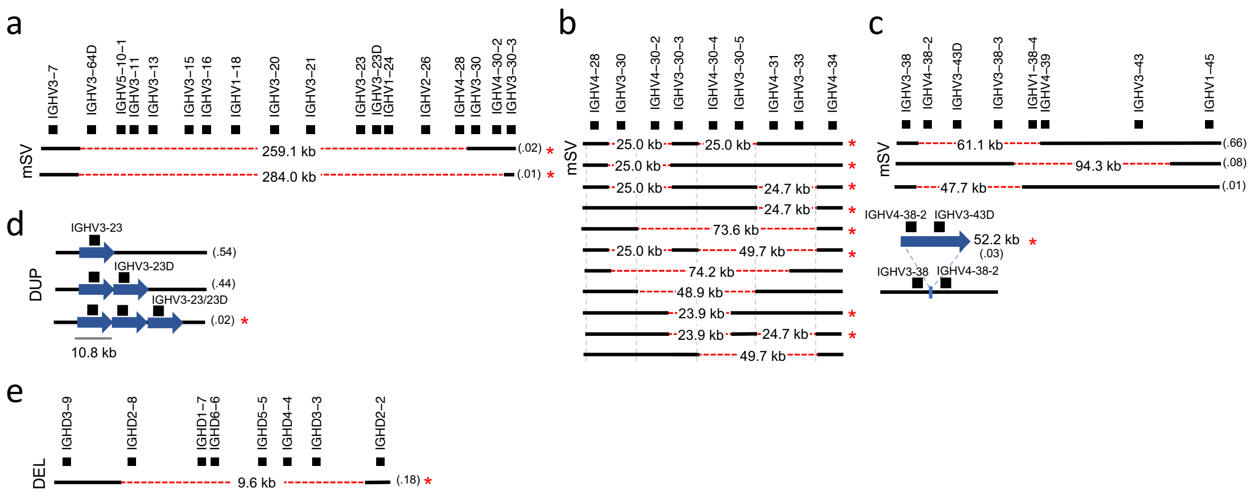

Supplementary Figure 3. Large SVs with novel SV alleles.

(a) A multi-allelic structural variant (mSV) with three alleles, including the reference assembly allele. Two of the SV alleles represent 259.1 and 284.9 Kbp deletions, deleting up to 16 genes. **(b)** mSV with 12 alleles. **(c)** mSV with 5 alleles: reference allele, 3 deletions and 1 insertion representing a partial duplication relative to the reference. **(d)** Duplication SV including alleles harboring 1 to 3 copies of the *IGHV3-23* gene. **(e)** Deletion in the IGHD gene region that deletes 6 IGHD genes. Red asterisks (**a-e**) indicate SV alleles that were not previously resolved at the genomic level.

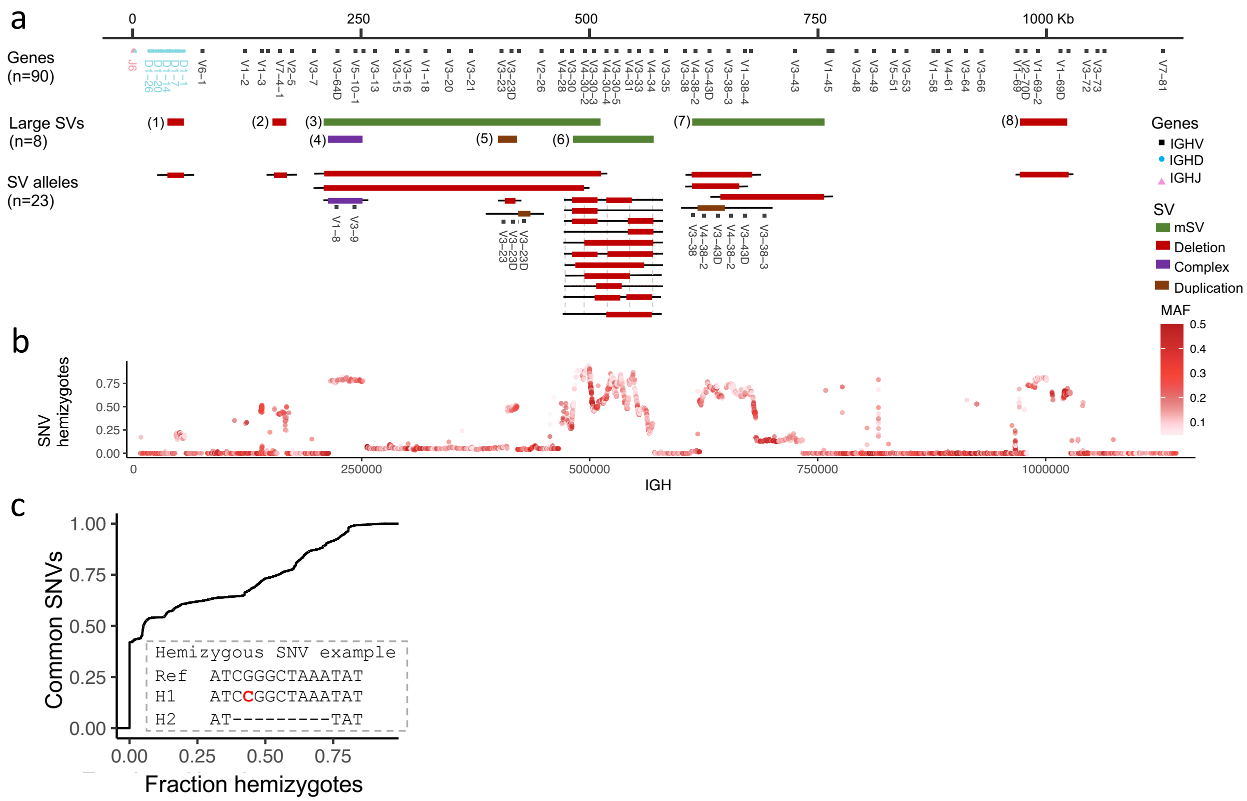

Supplementary Figure 4. SNVs genotyped as hemizygous.

(a) Identical figure as Figure 1a, replicated here for context. **(b)** Fraction of hemizygotes across all common single nucleotide variants (SNVs) color coded by minor allele frequency. **(c)** The fraction of hemizygotes across all common SNVs. The embedded panel is an example of a hemizygous SNV.

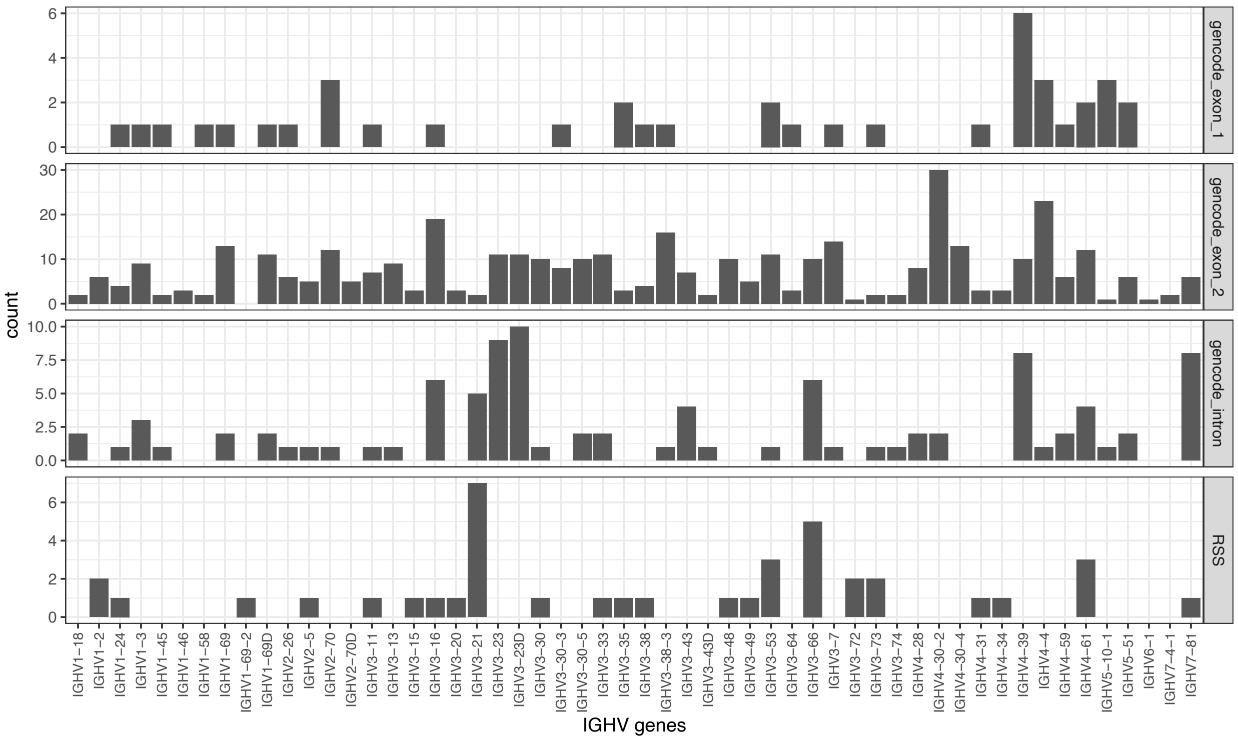

Supplementary Figure 5. Number of SNVs in different gene components across all IGHV genes.

For each IGHV gene, the number of SNVs identified in exons, introns and RSSs are shown.

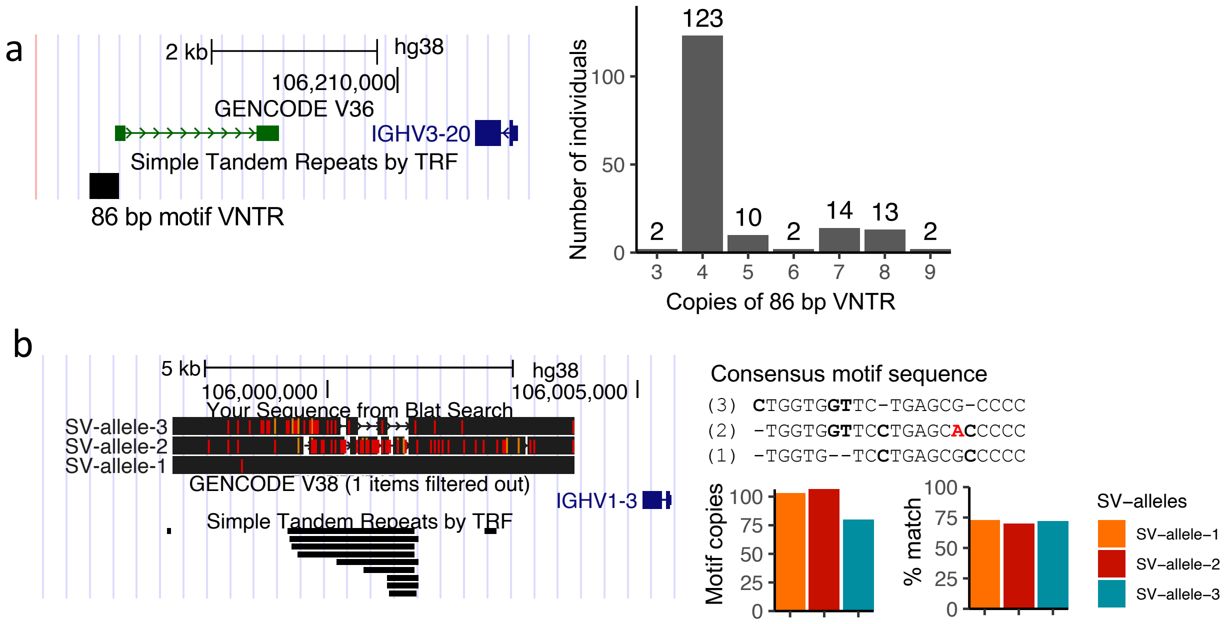

Supplementary Figure 6. Examples of polymorphic indels and small SVs.

(a) A polymorphic 86 bp variable number tandem repeat (VNTR) upstream of *IGHV3-20*. Across the cohort most individuals have 4 copies of the motif, however, some individuals have up to 9 copies. **(b)** A complex SV upstream of *IGHV1-3* is most likely derived from a degenerate tandem repeat. Three different SV alleles were identified with different motif sequences. The number of motif copies differed between SV alleles. An alignment of the consensus motif sequence to all the motifs in the SV alleles showed low sequence identity.

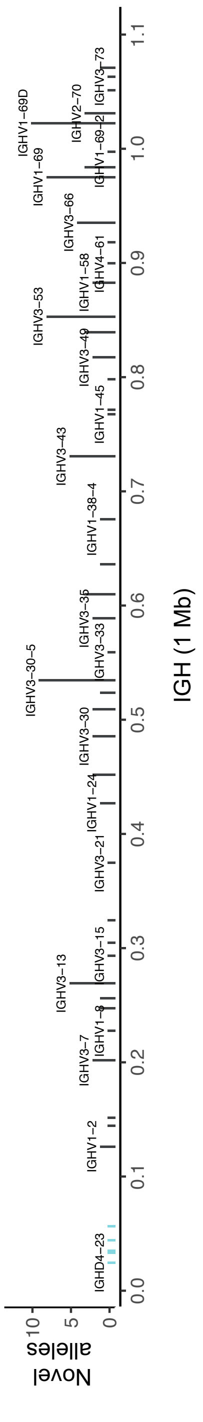

Supplementary Figure 7. Number of novel alleles per gene.

Each line is a gene. The x-axis are the genomic coordinates of the gene and y-axis displays the number of novel alleles per gene. Blue lines correspond to IGHD genes, and black lines correspond to IGHV genes.

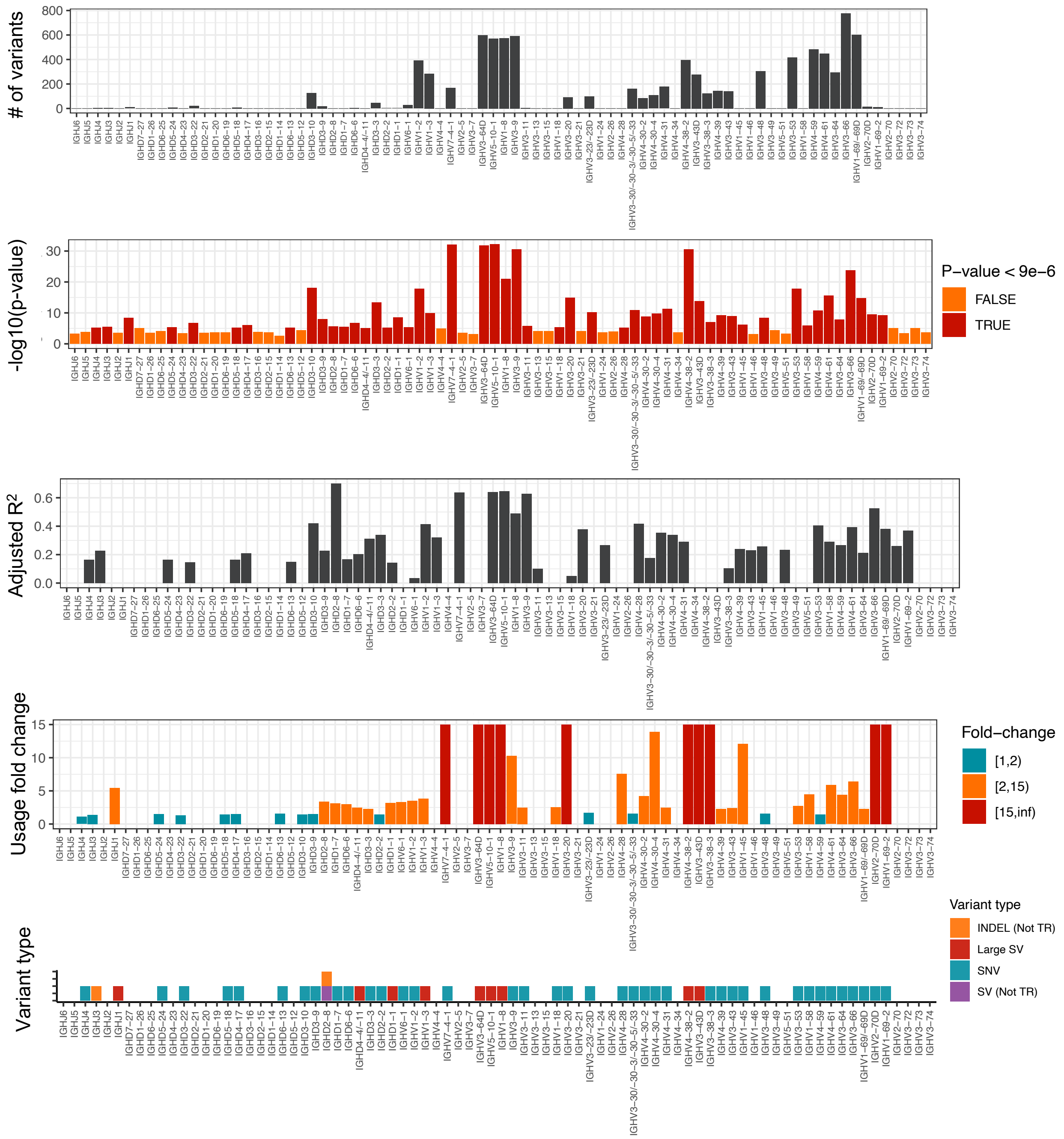

Supplementary Figure 8. Gene usage QTL statistics for the IgG repertoire.

The five panels show (1) number of associated variant using ANOVA and linear regression (*P value* < 9e-6 threshold after Bonferroni correction) and (2) the *P value*, (3) adjusted R^2 for

variance in gene usage explained, (4) fold change between genotypes and (5) the variant type for the lead guQTL.

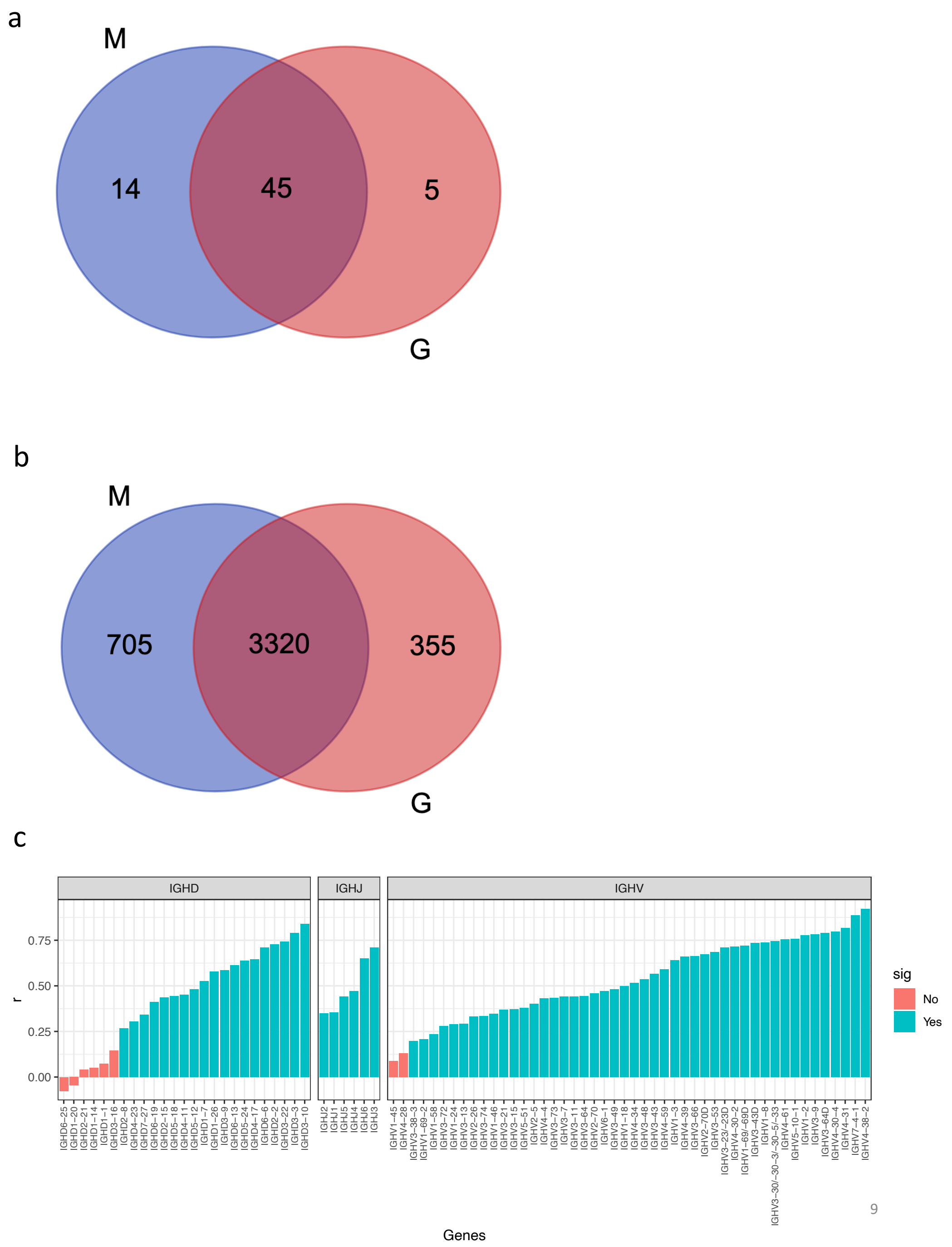

Supplementary Figure 9. Comparison of the IgM and IgG gene usage association results.

(a) The overlap of gene usage QTL genes between IgM and IgG. **(b)** The overlap of gene usage QTL variants between IgM and IgG. **(c)** Gene usage correlation between the IgM and IgG repertoire.

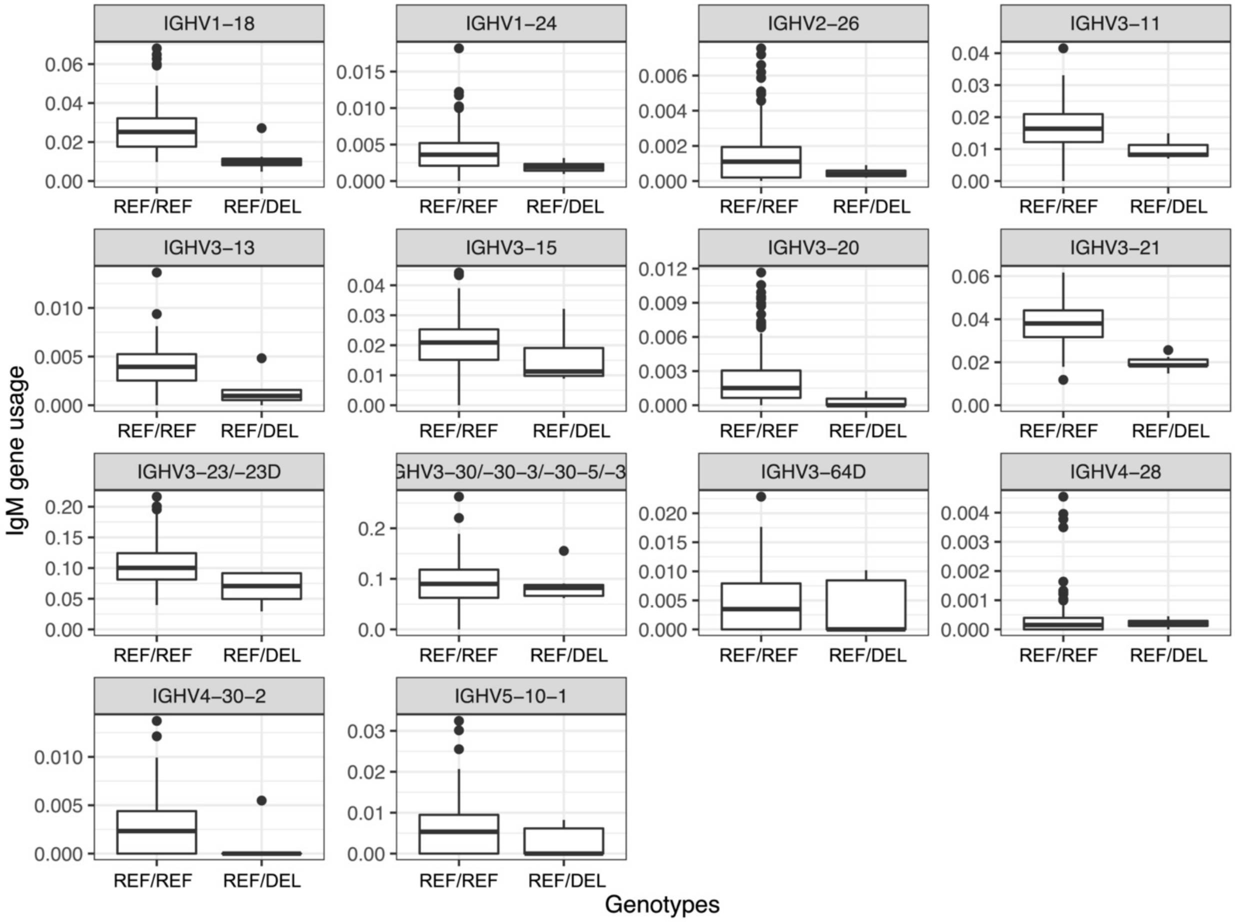

Supplementary Figure 10. Gene usage for genes in the largest deletion identified.

Individuals carrying the deletion (n=7 individuals) had overall lower usage of deleted genes than individuals that did not carry the deletion (n=147 individuals). Boxplots display the median, 25th percentile, 75th percentile, and whiskers that extend up to 1.5 times the inter-quartile range (IQR) from the respective percentiles. Any data points outside the whiskers are also plotted.

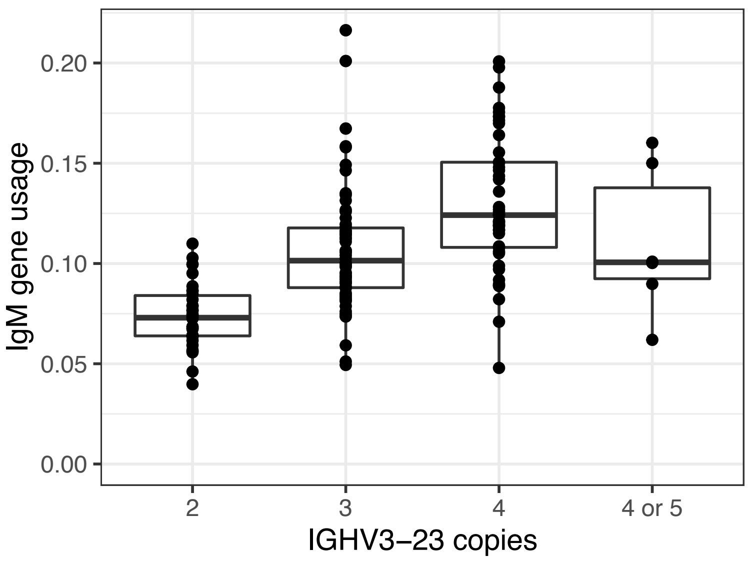

Supplementary Figure 11. Gene usage for individuals with variable *IGHV3-23* gene copy number.

IGHV3-23 is affected by a DUP SV and therefore the number of *IGHV3-23* copies varies between individuals. The boxplot shows variation in gene usage between individuals with different *IGHV3-23* diploid copy number. Individuals were grouped in cases for which copy number (either “4” of “5” copies) could not be determined. The number of individuals with 2, 3 4 and 4 or 5 copies is 34, 58, 46 and 6, respectively.

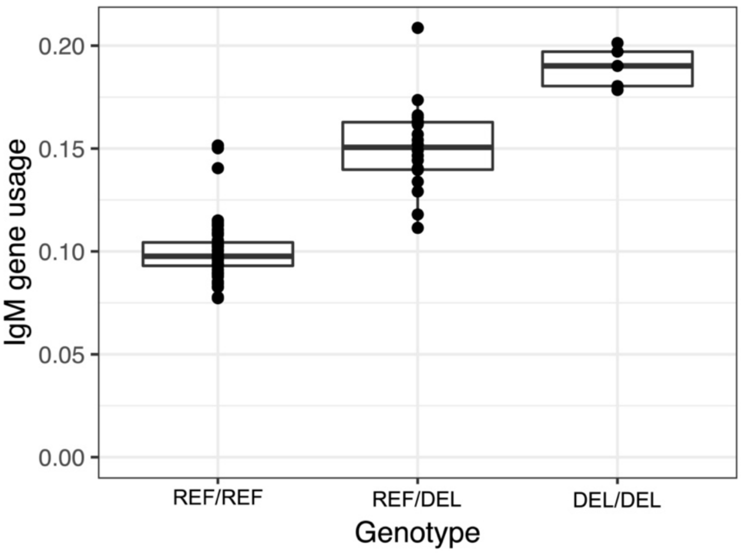

Supplementary Figure 12. Gene usage for *IGHD3-10* is associated with the IGHD gene region deletion.

IGHD3-10 is outside of the deletion (SV (1) in Fig. 1a) in the IGHD gene region, but its usage is associated with the deletion genotype. The number of individuals with a REF/REF, REF/DEL and DEL/DEL genotype is 51, 18, and 5, respectively. Boxplots display the median, 25th percentile, 75th percentile, and whiskers that extend up to 1.5 times the inter-quartile range (IQR) from the respective percentiles. Any data points outside the whiskers are also plotted.

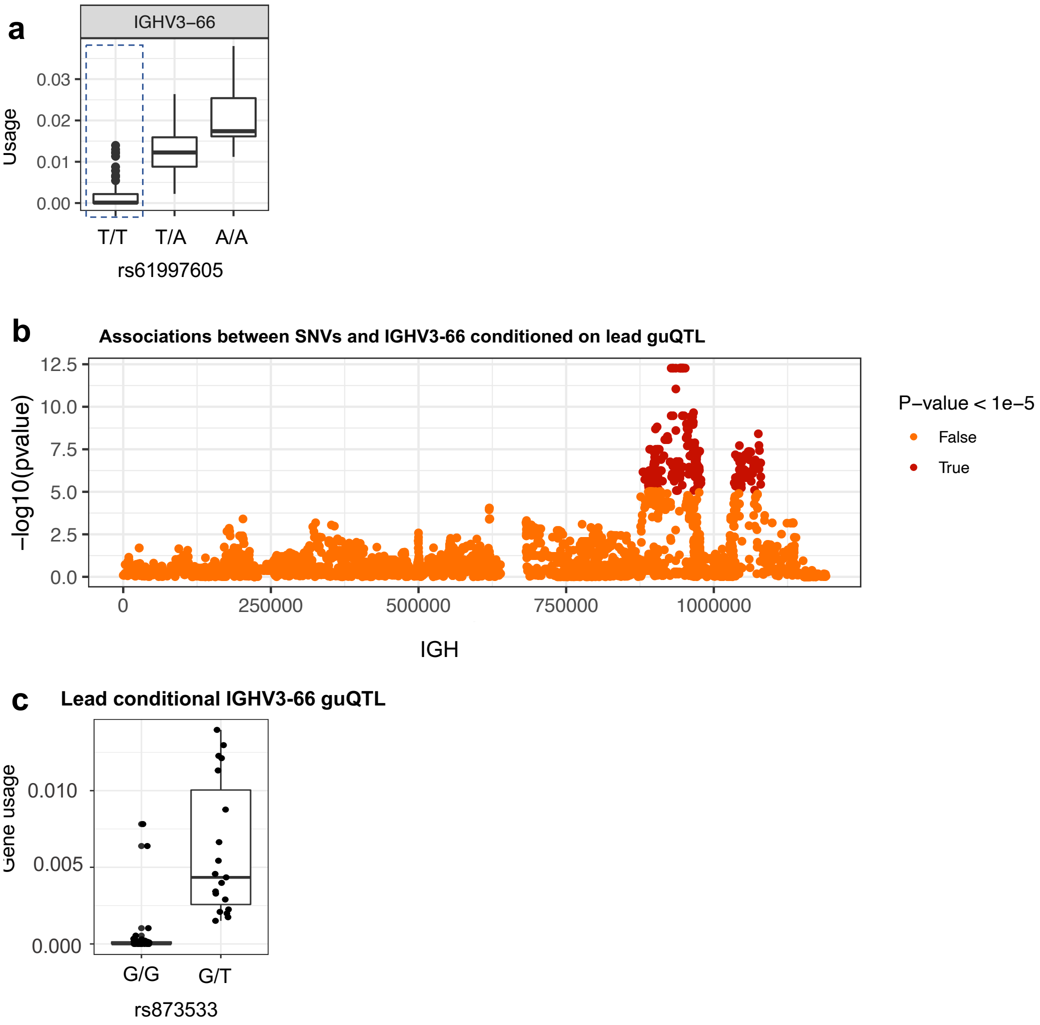

Supplementary Figure 13. Example of guQTL conditional analysis for the gene *IGHV3-66*.

(a) Boxplot showing gene usage variation for *IGHV3-66*, partitioned by genotypes at the lead guQTL. **(b)** Conditional analysis for *IGHV3-66* finds additional variants (*P value* = 5.38E-13) associated with gene usage (linear regression; $P < 1e\text{-}5$ threshold after Bonferroni correction). Manhattan plot showing the statistical significance of all SNVs tested for secondary effects on gene usage (red indicates Bonferroni corrected significant SNVs), after selecting individuals from a single genotype group from the original lead guQTL (blue dotted box, panel a). **(c)** Boxplot showing gene usage variation partitioned by genotypes at the secondary guQTL.

Boxplots display the median, 25th percentile, 75th percentile, and whiskers that extend up to 1.5 times the inter-quartile range (IQR) from the respective percentiles. Any data points outside the whiskers are also plotted.

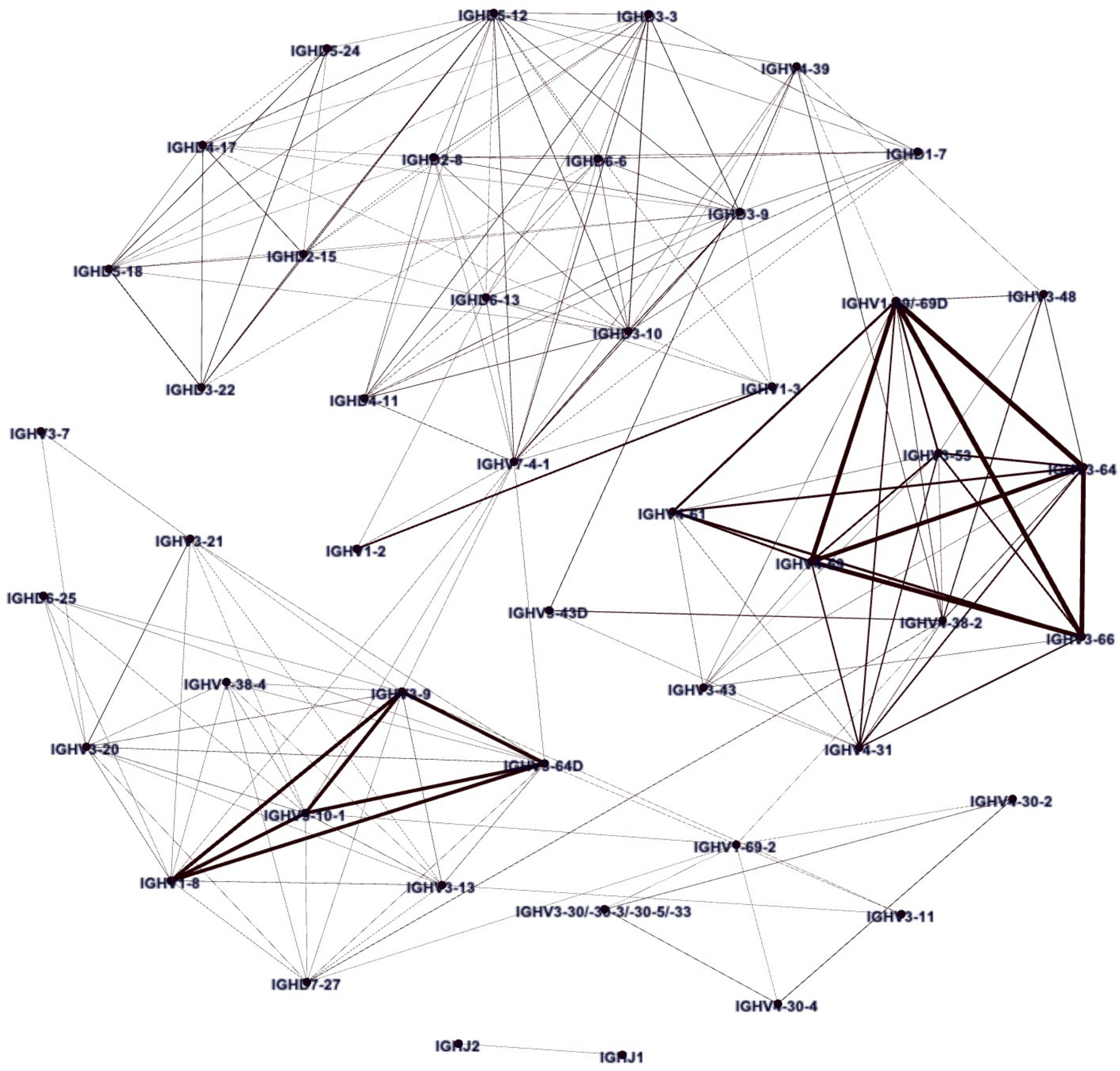

Supplementary Figure 14. Network of all genes connected by shared guQTLs.

In the network, nodes represent genes, and edges connect two nodes (genes) if both have at least one overlapping guQTL. This was performed for all genes based on IgM guQTL analysis results.

a

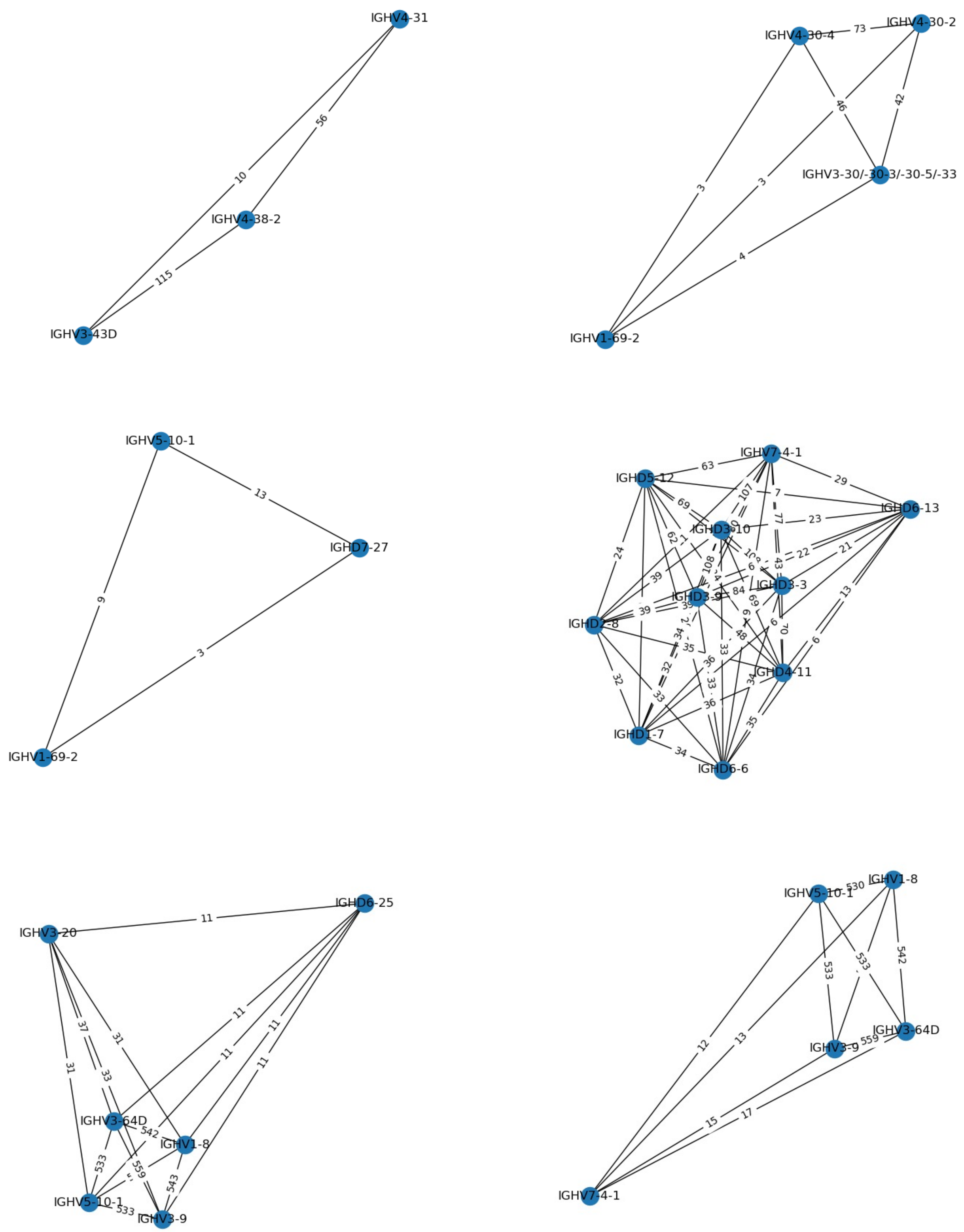
IGHV4-31
IGHV4-38-2
IGHV3-43D
10
56
115
IGHV4-30-4
IGHV4-30-2
IGHV3-30/-30-3/-30-5/-33
IGHV1-69-2
73
46
42
3
3
4
IGHV5-10-1
IGHD7-27
IGHV1-69-2
13
9
3
IGHV7-4-1
IGHD5-12
IGHD6-13
IGHD3-10
IGHD3-3
IGHD3-9
IGHD2-8
IGHD4-11
IGHD1-7
IGHD6-6
IGHV3-20
IGHD6-25
IGHV3-64D
IGHV1-8
IGHV5-10-1
IGHV3-9
IGHV5-10-1
IGHV1-8
IGHV3-64D
IGHV3-9
IGHV7-4-1

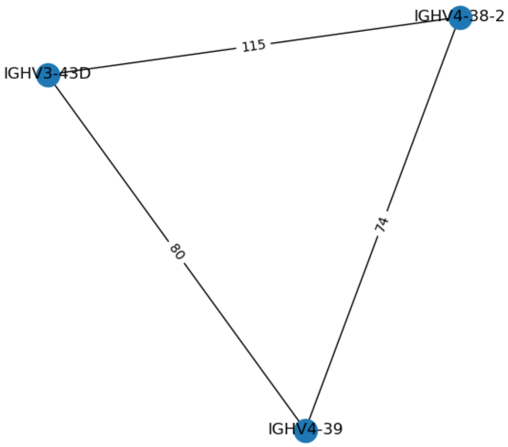
IGHV4-38-2
IGHV3-43D
IGHV4-39
115
80
74

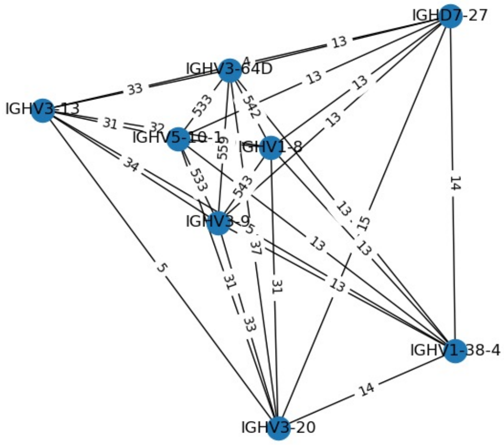
IGHD7-27
IGHV3-64D
IGHV3-13
IGHV5-10-1
IGHV1-8
IGHV3-9
IGHV1-38-4
IGHV3-20

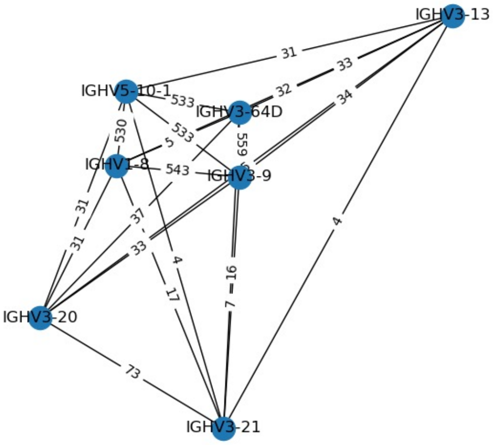
IGHV3-13
IGHV5-10-1
IGHV3-64D
IGHV1-8
IGHV3-9
IGHV3-20
IGHV3-21

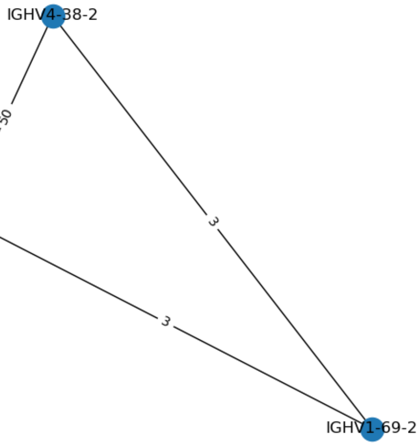
IGHV4-38-2
IGHD7-27
IGHV1-69-2
50
3
3

b

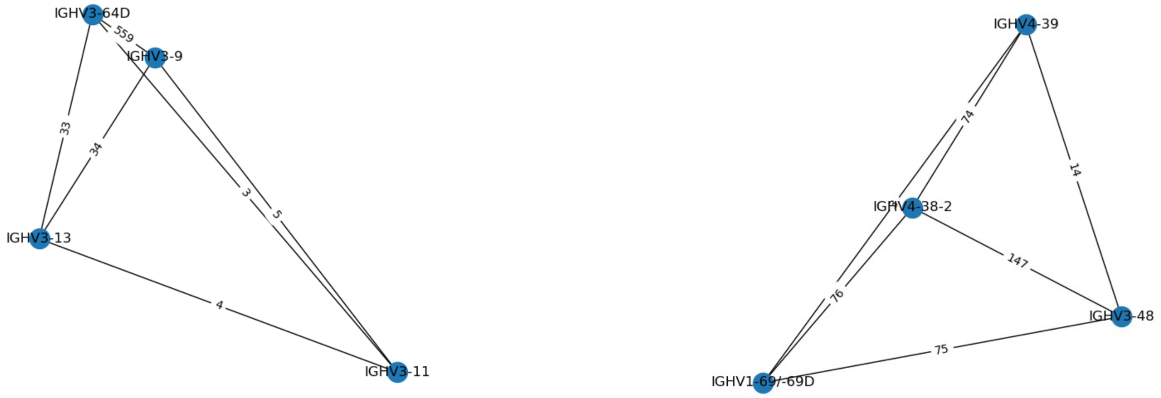
IGHV3-64D
IGHV3-9
IGHV3-13
IGHV3-11
559
33
34
3
5
4
IGHV4-39
IGHV4-38-2
IGHV3-48
IGHV1-69/-69D
74
14
147
76
75

c

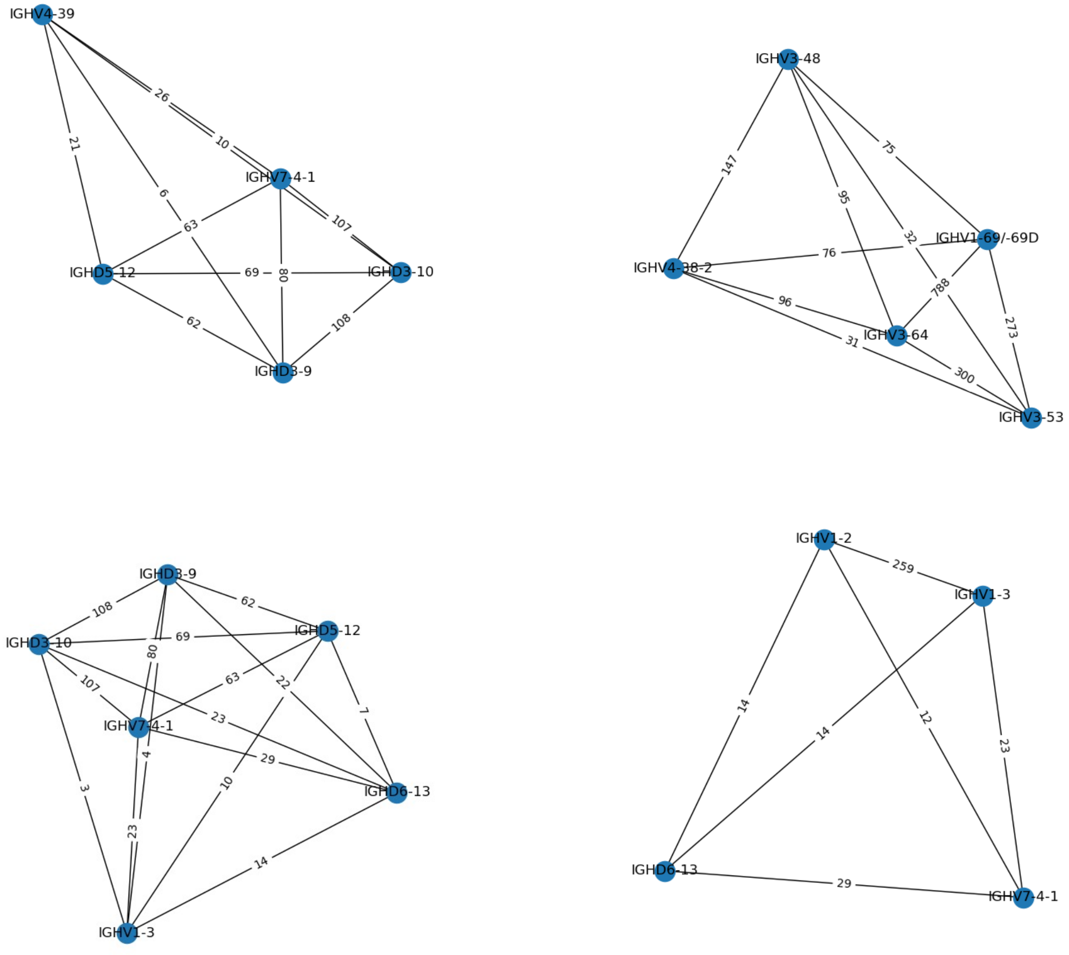
IGHV4-39
IGHV7-4-1
IGHD5-12
IGHD3-10
IGHD3-9
26
10
21
6
63
107
69
80
62
108
IGHV3-48
IGHV1-69/-69D
IGHV4-38-2
IGHV3-64
IGHV3-53
147
75
95
32
76
788
96
273
31
300
IGHD3-9
IGHD3-10
IGHD5-12
IGHV7-4-1
IGHD6-13
IGHV1-3
108
62
69
80
107
63
22
23
7
4
29
3
10
23
14
IGHV1-2
IGHV1-3
IGHD6-13
IGHV7-4-1
259
14
14
12
23
29

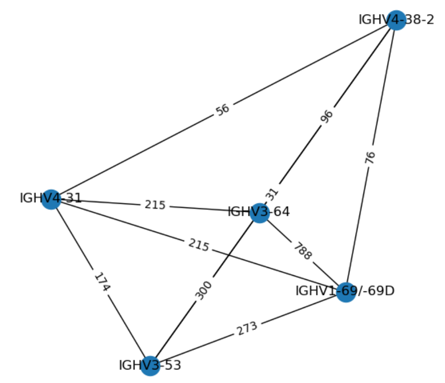
IGHV4-38-2
IGHV4-31
IGHV3-64
IGHV1-69/-69D
IGHV3-53

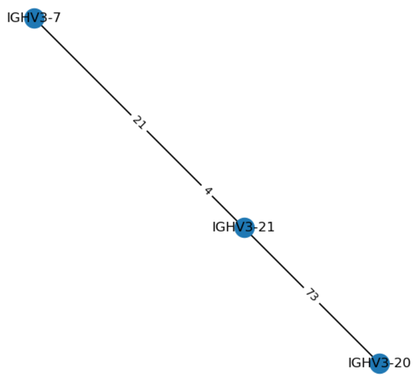
IGHV3-7
IGHV3-21
IGHV3-20

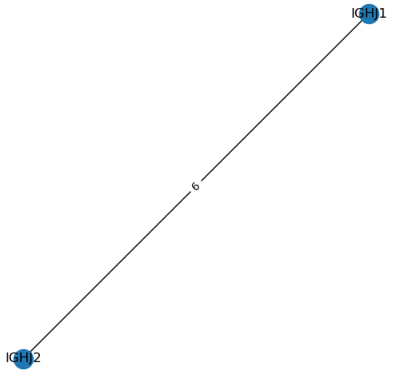
IGHJ1
IGHJ2

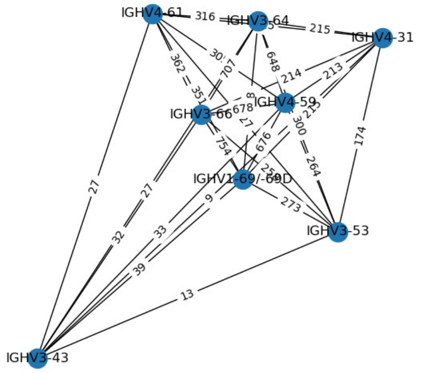
IGHV4-61
IGHV3-64
IGHV4-31
IGHV4-59
IGHV3-66
IGHV1-69/-69D
IGHV3-53
IGHV3-43

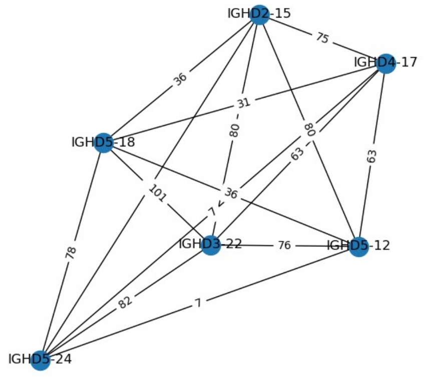
IGHD2-15
IGHD4-17
IGHD5-18
IGHD3-22
IGHD5-12
IGHD5-24

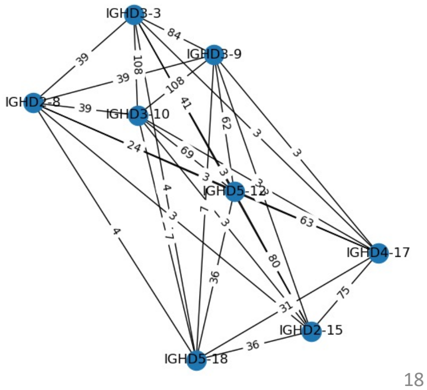
IGHD3-3
IGHD3-9
IGHD2-8
IGHD3-10
IGHD5-12
IGHD4-17
IGHD2-15
IGHD5-18

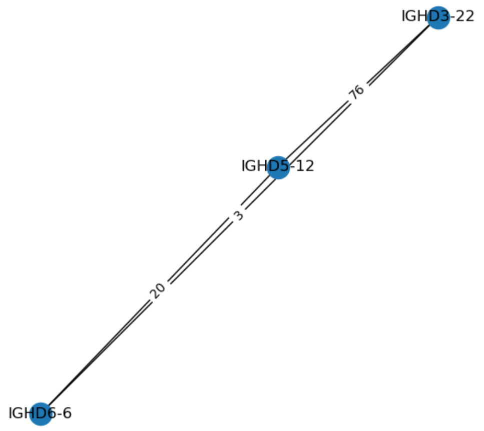

Supplementary Figure 15. Cliques found in the network.

(a) Cliques represented predominately by genes within SVs; **(b)** two cliques represented by genes that are both directly and indirectly impacted SVs; and **(c)** cliques represented by genes primarily associated with SNVs, rather than SVs.

Supplementary References

1. Watson, C. T. *et al.* Complete haplotype sequence of the human immunoglobulin heavy-chain variable, diversity, and joining genes and characterization of allelic and copy-number variation. *Am. J. Hum. Genet.* **92**, 530–546 (2013).
2. Kirik, U., Greiff, L., Levander, F. & Ohlin, M. Parallel antibody germline gene and haplotype analyses support the validity of immunoglobulin germline gene inference and discovery. *Mol. Immunol.* **87**, 12–22 (2017).
3. Watson, C. T. *et al.* Complete haplotype sequence of the human immunoglobulin heavy-chain variable, diversity, and joining genes and characterization of allelic and copy-number variation. *Am. J. Hum. Genet.* **92**, 530–546 (2013).
4. Matsuda, F. *et al.* The complete nucleotide sequence of the human immunoglobulin heavy chain variable region locus. *J. Exp. Med.* **188**, 2151–2162 (1998).
5. Sasso, E. H., Buckner, J. H. & Suzuki, L. A. Ethnic differences of polymorphism of an immunoglobulin VH3 gene. *J. Clin. Invest.* **96**, 1591–1600 (1995).
6. Kidd, M. J., Jackson, K. J. L., Boyd, S. D. & Collins, A. M. DJ Pairing during VDJ Recombination Shows Positional Biases That Vary among Individuals with Differing IGHD Locus Immunogenotypes. *J. Immunol.* **196**, 1158–1164 (2016).
7. Kidd, M. J. *et al.* The inference of phased haplotypes for the immunoglobulin H chain V region gene loci by analysis of VDJ gene rearrangements. *J. Immunol.* **188**, 1333–1340 (2012).
8. Tonegawa, S. Somatic Generation of Antibody Diversity. *Immunology* 145–162 (1995) doi:10.1016/b978-012274020-6/50014-3.

9. Xu, J. L. & Davis, M. M. Diversity in the CDR3 Region of VH Is Sufficient for Most Antibody Specificities. *Immunity* vol. 13 37–45 (2000).
