# Supplementary material for: Genetic variation in the immunoglobulin heavy chain locus shapes the human antibody repertoire
Source: Nat Commun. 2023 Jul 21;14:4419. doi: 10.1038/s41467-023-40070-x (PMC10362067; doi:10.1038/s41467-023-40070-x)
Supplement: Supplementary file 3 — Description of Additional Supplementary Files [file 41467_2023_40070_MOESM3_ESM.pdf]

Description of Additional Supplementary Files

File Name: Supplementary Data 1
Description: Sample information with details regarding AIRR-seq and Pacific BioScience datasets

File Name: Supplementary Data 2
Description: Description of large IGH SVs

File Name: Supplementary Data 3
Description: Description of novel alleles

File Name: Supplementary Data 4
Description: Lead guQTL results

File Name: Supplementary Data 5
Description: Conditional guQTL results

File Name: Supplementary Data 6
Description: Statistics for allele distribution differences between donors partitioned by guQTL genotype

File Name: Supplementary Data 7
Description: IGHV and IGHC primers for AIRR-seq
