# Supplementary material for: Genetic variation in the immunoglobulin heavy chain locus shapes the human antibody repertoire
Source: Nat Commun. 2023 Jul 21;14:4419. doi: 10.1038/s41467-023-40070-x (PMC10362067; doi:10.1038/s41467-023-40070-x)
Supplement: Supplementary file 5 — Reporting Summary [file 41467_2023_40070_MOESM5_ESM.pdf]

nature portfolio

Corresponding author(s): Corey T. Watson; Wayne A. Marasco; Melissa L. Smith

Last updated by author(s): 06/16/2023

Reporting Summary

Nature Portfolio wishes to improve the reproducibility of the work that we publish. This form provides structure for consistency and transparency in reporting. For further information on Nature Portfolio policies, see our Editorial Policies and the Editorial Policy Checklist.

Statistics

For all statistical analyses, confirm that the following items are present in the figure legend, table legend, main text, or Methods section.

n/a	Confirmed	
☐	☒	The exact sample size (n) for each experimental group/condition, given as a discrete number and unit of measurement
☐	☒	A statement on whether measurements were taken from distinct samples or whether the same sample was measured repeatedly
☐	☒	The statistical test(s) used AND whether they are one- or two-sided<br>*Only common tests should be described solely by name; describe more complex techniques in the Methods section.*
☐	☒	A description of all covariates tested
☐	☒	A description of any assumptions or corrections, such as tests of normality and adjustment for multiple comparisons
☐	☒	A full description of the statistical parameters including central tendency (e.g. means) or other basic estimates (e.g. regression coefficient) AND variation (e.g. standard deviation) or associated estimates of uncertainty (e.g. confidence intervals)
☐	☒	For null hypothesis testing, the test statistic (e.g. F, t, r) with confidence intervals, effect sizes, degrees of freedom and P value noted<br>*Give P values as exact values whenever suitable.*
☒	☐	For Bayesian analysis, information on the choice of priors and Markov chain Monte Carlo settings
☒	☐	For hierarchical and complex designs, identification of the appropriate level for tests and full reporting of outcomes
☐	☒	Estimates of effect sizes (e.g. Cohen's d, Pearson's r), indicating how they were calculated

Our web collection on statistics for biologists contains articles on many of the points above.

Software and code

Policy information about availability of computer code

Data collection	- For collection of genomic data, we utilized our published/custom pipeline, IGenotyper v1.01 (10.5281/zenodo.7968412). All code and associated files are available on github: https ://github.com/oscarlr/IGenotyper.<br>- IGenotyper integrates several published and open source software packages: BLASR (5.3.3=h018d624_2) , WhatsHap (1.1.dev78+g8f4c0c0), MsPAC (https://github.com/oscarlr/MsPac; commit 3a741dc) and Canu (2.0=he1b5a44_0).<br>- Downstream genetic variant discovery and analysis also leveraged bcftools (v1.16), BEAGLE (v228Jun21.220), Tandem Repeats Finder (v4.09.1), and PacMonSTR (https://github.com/oscarlr-TRs/PacMonSTR; 10.5281/zenodo.7968464).<br>- For adaptive immune receptor repertoire sequencing data processing and analysis, we used tools and packages from the Immcantation (v4.4.0) pipeline: https://immcantation.readthedocs.io/en/stable/. This pipeline integrates a suite of open source software and functions. Specifically, we utilized pRESTO (v0.7.1) and Change-o (v1.3.0).<br>- ComBat-Seq (https://github.com/zhangyuqing/ComBat-seq; commit 2673dd8) was used to normalize read counts.
Data analysis	The networkx (release 3.1) python library (networkx.org) was used to create gene by variant networks.

For manuscripts utilizing custom algorithms or software that are central to the research but not yet described in published literature, software must be made available to editors and reviewers. We strongly encourage code deposition in a community repository (e.g. GitHub). See the Nature Portfolio guidelines for submitting code & software for further information.

Data

Policy information about availability of data

All manuscripts must include a data availability statement. This statement should provide the following information, where applicable:

- Accession codes, unique identifiers, or web links for publicly available datasets
- A description of any restrictions on data availability
- For clinical datasets or third party data, please ensure that the statement adheres to our policy

The IGH locus long-read sequencing data and AIRR-seq datasets generated in this study have been deposited in the BioProject repository PRJNA555323 [https://www.ncbi.nlm.nih.gov/bioproject/?term=PRJNA555323]. The study utilized the following public datasets: (1) reference genome from https://github.com/oscarlr/IGenotyper, (2) IMGT database (release 201130-2) from https://www.imgt.org/download/V-QUEST/, (3) Encode candidate regulatory regions from http://hgdownload.soe.ucsc.edu/gbdb/hg38/encode3/ccre/, and (4) GWAS catalog from https://www.ebi.ac.uk/gwas/api/search/downloads/full.

Research involving human participants, their data, or biological material

Policy information about studies with human participants or human data. See also policy information about sex, gender (identity/presentation), and sexual orientation and race, ethnicity and racism.

Reporting on sex and gender	Sex was not available for all of the participants studied here. It is reported in sample metadata files when available.
Reporting on race, ethnicity, or other socially relevant groupings	Self-reported ethnicity was not used in this study as a covariate. It is, however, reported as sample metadata.
Population characteristics	Samples in the cohort ranged in age from 17 to 78 years (mean = 42) and included individuals who self-reported as White (n=81), South Asian (n=20), Black or African American (n=19), Hispanic or Latino (n=19), East Asian (n=11), Native Hawaiian or Other Pacific Islander (n=1), American Indian or Alaska Native (n=1), or unknown (n=2).
Recruitment	Samples were recruited as part of previous studies or purchased through commercial vendors. No specific criteria were used to guide recruitment.
Ethics oversight	This study complies with all relevant ethical regulations. The study and protocol were reviewed and approved by the Dana-Farber Cancer Institute (DFCI), Stanford University and University of Louisville Institutional Review Boards (IRBs). Informed consent for study participation and collection of blood samples was obtained with research volunteers signing a consent form approved by the DFCI IRB.

Note that full information on the approval of the study protocol must also be provided in the manuscript.

Field-specific reporting

Please select the one below that is the best fit for your research. If you are not sure, read the appropriate sections before making your selection.

☒ Life sciences ☐ Behavioural & social sciences ☐ Ecological, evolutionary & environmental sciences

For a reference copy of the document with all sections, see nature.com/documents/nr-reporting-summary-flat.pdf

Life sciences study design

All studies must disclose on these points even when the disclosure is negative.

Sample size	The sample size was 154. The sample size was based on sample availability, affording the study sufficient sample size to identify large genetic effects of common genetic variants.
Data exclusions	Single nucleotide variants (SNVs) with a Hardy Weinberg Equilibrium (HWE) p value less than 0.000001 were filtered using bcftools. SNVs found in less than 5 individuals were removed if they did not have HiFi read support. The SNVs passing these stringent quality control thresholds were used to impute missing genotypes using Beagle (v228Jun21.220). The resulting SNVs were again filtered if they contained a HWE value less 0.000001. Common SNVs were selected if they were genotyped in at least 40 individuals and had a MAF equal to or greater than 0.05. The same criteria were applied to SNVs selected for conditional analysis.<br>All structural variants (SVs) were genotyped using IGenotyper and manually inspected using the integrative genomics viewer (IGV; https://software.broadinstitute.org/software/igv/) . SVs with a MAF less than 0.05 were not included in the guQTL analysis. SVs that could not be resolved using HiFi reads or assemblies were not genotyped and were not included in downstream analyses.
Replication	Given the technical complexity of the datasets generated (they are the first of their kind), replication in a second cohort was not feasible in the current study.
Randomization	All individuals were grouped as "healthy donors". Statistical models included "age" and "adaptive immune receptor repertoire sequencing method" as covariables, as both have been shown to be relevant to variation observed in expressed repertoire datasets.

Blinding	Blinding was not relevant to our study. No phenotypes for which blinding would be appropriate were investigated here.<br><br>Expression Quantitative trait locus (eQTL) studies are not typically conducted in a blinded manner for several reasons:<br>1 Discovery-based research: in eQTL studies, researchers aim to investigate associations of specific genetic variants and gene expression in an unbiased fashion. Blinding is more commonly employed in experimental designs where bias and subjectivity can impact the results, such as in clinical trials. In eQTL studies, the focus is on identifying specific genetic variants and their effects, rather than subjective interpretations.<br>2 Data-driven analyses: eQTL studies involve analysis of large-scale genomic data. The analyses are often conducted using computational methods and statistical models, where the emphasis is on data analysis techniques rather than subjective interpretation by the researchers.<br>3 Reproducibility and transparency: eQTL studies strive to ensure reproducibility and transparency in their methodologies and analyses. Blinding may introduce additional complexities and potentially confound the interpretation of results. By conducting analyses in an open and transparent manner, other researchers can replicate and validate the findings, which strengthens the scientific rigor of the study.<br>4 Control for confounding factors: eQTL studies typically incorporate appropriate statistical models to control for potential confounding factors, such as batch effects, population structure, and technical artifacts. By implementing these controls, researchers aim to minimize the influence of extraneous variables and enhance the accuracy and reliability of the results.

Reporting for specific materials, systems and methods

We require information from authors about some types of materials, experimental systems and methods used in many studies. Here, indicate whether each material, system or method listed is relevant to your study. If you are not sure if a list item applies to your research, read the appropriate section before selecting a response.

Materials & experimental systems

n/a	Involved in the study
☒	☐ Antibodies
☒	☐ Eukaryotic cell lines
☒	☐ Palaeontology and archaeology
☒	☐ Animals and other organisms
☒	☐ Clinical data
☒	☐ Dual use research of concern
☒	☐ Plants

Methods

n/a	Involved in the study
☒	☐ ChIP-seq
☒	☐ Flow cytometry
☒	☐ MRI-based neuroimaging
